# Randomised, controlled trial of N-acetylcysteine for treatment of acute exacerbations of chronic obstructive pulmonary disease [ISRCTN21676344]

**DOI:** 10.1186/1471-2466-4-13

**Published:** 2004-12-06

**Authors:** Peter N Black, Althea Morgan-Day, Tracey E McMillan, Phillippa J Poole, Robert P Young

**Affiliations:** 1 Department of Medicine, University of Auckland, Private Bag 92019, Auckland, New Zealand

## Abstract

**Background:**

Prophylactic treatment with N-acetylcysteine (NAC) for 3 months or more is associated with a reduction in the frequency of exacerbations of chronic obstructive pulmonary disease (COPD). This raises the question of whether treatment with NAC during an acute exacerbation will hasten recovery from the exacerbation.

**Methods:**

We have examined this in a randomised, double-blind, placebo controlled trial. Subjects, admitted to hospital with an acute exacerbation of COPD, were randomised within 24 h of admission to treatment with NAC 600 mg b.d. (n = 25) or matching placebo (n = 25). Treatment continued for 7 days or until discharge (whichever occurred first). To be eligible subjects had to be ≥ 50 years, have an FEV_1 _≤ 60% predicted, FEV_1_/VC ≤ 70% and ≥ 10 pack year smoking history. Subjects with asthma, heart failure, pneumonia and other respiratory diseases were excluded. All subjects received concurrent treatment with prednisone 40 mg/day, nebulised salbutamol 5 mg q.i.d and where appropriate antibiotics. FEV_1_, VC, SaO_2 _and breathlessness were measured 2 hours after a dose of nebulised salbutamol, at the same time each day. Breathlessness was measured on a seven point Likert scale.

**Results:**

At baseline FEV_1 _(% predicted) was 22% in the NAC group and 24% in the control group. There was no difference between the groups in the rate of change of FEV_1_, VC, SaO_2 _or breathlessness. Nor did the groups differ in the median length of stay in hospital (6 days for both groups).

**Conclusions:**

Addition of NAC to treatment with corticosteroids and bronchodilators does not modify the outcome in acute exacerbations of COPD.

## Background

Exacerbations are an important cause of morbidity in Chronic Obstructive Pulmonary Disease. Seemungal et al found that exacerbations were an important determinant of quality of life in COPD [1]. In addition hospital admissions with exacerbations account for a large proportion of the expenditure on the treatment of COPD [2]. This has led to a search for strategies to prevention exacerbations and to hasten their resolution when they do occur. A systematic review found that treatment with mucolytics for 2 months or more reduced the frequency of exacerbations by 29% [3]. The majority of the studies included in the review were with N-acetylcysteine. These findings are supported by a recent pharmacoepidemiologic study [4]. Aside from its action as a mucolytic, N-acetylcysteine is an antioxidant [5,6] and has anti-inflammatory actions [7] and this could contribute to its actions in preventing exacerbations of COPD. Exacerbations of COPD are characterized by increased infiltration of the airways with neutrophils and eosinophils [8] and by increased production of reactive oxygen species [9]. Reactive oxygen species can activate the epidermal growth factor receptor to promote mucus secretion [10] and this is one of the potential mechanisms by which an increase in reactive oxygen species could lead to a worsening of an acute exacerbation. In view of this we wondered whether N-acetylcysteine might also be useful in the treatment of patients presenting with an acute exacerbation of COPD. We undertook a randomised, double-blind, placebo-controlled, parallel group study of oral N-acetylcysteine 600 mg b.d. in addition to standard treatment in patients admitted to hospital with an acute exacerbation of COPD.

## Methods

Patients were eligible for inclusion in the study if they had a physician diagnosis of COPD, were ≥ 50 years of age, had a smoking history ≥ 10 pack years and had been admitted to hospital with an acute exacerbation of their COPD in the previous 24 hours. In addition they were required to have FEV_1 _≤ 60% predicted and FEV_1_/VC ≤ 0.7 at time of inclusion. Patients were excluded if they had any of the following conditions: asthma (as the primary diagnosis), heart failure, bronchiectasis, bronchial carcinoma, interstitial lung disease, pneumonia. They were also excluded if they were unable to comply with the study procedures because they did not speak English or were demented or if they had any other medical problems that in the opinion of the investigator would interfere with the conduct of the study.

Subjects were treated with N-acetylcysteine 600 mg twice daily (b.d.) or matching placebo. Treatment was continued for 7 days or until discharge whichever occurred first. Effervescent N-acetylcysteine tablets were purchased from Zambon pharmaceuticals (Milan, Italy) and were repackaged in size 0 gelatine capsules. Each capsule contained 300 mg of N-acetylcysteine. Placebo capsules were prepared containing lactose as a filler. A randomisation schedule was drawn up by the hospital pharmacy and patients were allocated sequential randomisation numbers as they entered the study.

In addition to N-acetylcysteine the patients received standard treatment for their exacerbation as specified by the hospital guidelines. This was oxygen therapy, prednisone 40 mg o.d. for one week and nebulised bronchodilators i.e. salbutamol 5 mg four times daily (q.i.d.) and ipratropium 0.5 mg q.i.d. Antibiotics were prescribed if the patients had increased volume and/or purulence of sputum. Mucolytics were not permitted during the trial except as study medicine.

The primary endpoint was breathlessness measured on a seven point Likert scale (Table 1). Secondary endpoints were FEV_1_, oxygen saturation and length of hospital stay. The study assessments were performed at the same time each day and two hours after the last dose of nebulised bronchodilator. Breathlessness was assessed prior to spirometry. Spirometry was performed according to American Thoracic Society criteria using a Vitalograph spirometer. Oxygen saturation was measured using a Nelcor N-20 pulse oximeter. Supplemental oxygen was stopped for 10 minutes before any of the measurements were performed. In addition the subjects were interrogated on each occasion about any possible adverse effects.

**Table 1:** 

	**Likert Scale for Breathlessness**
1	Extremely short of breath
2	Very short of breath
3	Quite a bit short of breath
4	Moderate shortness of breath
5	Some shortness of breath
6	A little shortness of breath
7	Not at all short of breath

The study was conducted at Auckland Hospital between June 2001 and October 2001. The study was approved by the Auckland Ethics Committee and all participants provided written informed consent.

### Power calculation

The power calculation was based on a previous study where we treated subjects for acute exacerbations of COPD with theophylline for up to seven days [11]. In that study the average improvement in Likert score from baseline to the end of the study was 1.95 with theophylline and 1.05 with placebo. We assumed the subjects in this study would have a similar distribution of Likert scores for breathlessness. On this basis, 50 participants gave us 88% power to detect a similar difference between the groups as in the previous study, at a 5% level of significance.

### Statistical analysis

The two treatment groups were compared at baseline using Student's t-test for normally distributed variables, Wilcoxon test for non-parametric data and Fisher's exact test for categorical data.

The effect of N-acetylcysteine on breathlessness, lung function and oxygen saturation was analysed by fitting the patient data to a random coefficient model using a mixed linear model approach (least squares regression). The baseline (Day 1) measurements were used as co-variates in the analysis. This allowed individual slopes and intercepts to be formed for each patient and their random variation incorporated in the model. This model adjusted for the varying number of observations available on the different patients. Non-normal dependent variables were rendered normal by transformation. Significant and main interaction effects were investigated by the method of Tukey.

All tests were two-tailed and a 5% significance level was maintained throughout these analyses. The analyses were carried out using SAS Version 8.0 (SAS Institute Inc, Cary, NC, USA.)

The life test procedure of SAS was used to compute nonparametric estimates of the length of stay function by the Kaplan-Meier method. Comparison between these functions was made using the Wilcoxon and log rank tests.

## Results

Two hundred and ten patients who had been admitted to hospital with an exacerbation of COPD were screened for the study. Fifty subjects were randomised to treatment with 25 subjects receiving N-acetylcysteine and 25 subjects receiving placebo. The commonest reasons for exclusion were concomitant heart failure (n = 41) or pneumonia (n = 35).

The two groups were similar at baseline in terms of age, smoking history, lung function, oxygen saturation and breathlessness (Table 2). There were more men in the placebo group but the difference was not statistically significant (p = 0.23). There was no difference between the groups in the use of inhaled bronchodilators prior to admission (Table 3). More subjects in the N-acetylcysteine group had been on treatment with inhaled corticosteroids prior to admission but this difference was not statistically significant (p = 0.16). Although we did not document the amount of sputum produced by the subjects most subjects presented both with an increased volume of sputum as well as breathlessness.

**Table 2:** Baseline characteristics of the subjects

	**N-Acetylcysteine **(n = 25)	**Placebo **(n = 25)
**Gender **Male/Female	11/14	19/6
**Age **Years (SD)	73.6 (7.8)	73.0 (8.2)
**Smoking History **Pack Years (SD)	44.4 (36.2)	53.7 (36.8)
**FEV_1 _**% predicted (SD)	22 (10)	24 (12)
**VC **% predicted (SD)	56 (18)	64 (22)
**SaO_2 _**% (SD)	90.2 (4.0)	90.4 (2.7)
**Breathlessness **Likert Score (IQ range)	4 (3–6)	4 (3–5)

Values are shown as mean and standard deviation except for Likert scores that are shown as median and interquartile range. There were no statistically significant differences between the two groups for any of the measures.

**Table 3:** Concurrent medications

	**N-acetylcysteine **(n = 25)	**Placebo **(n = 25)
Inhaled steroids	15	9
Oral prednisone	9	6
Short acting inhaled beta-agonists	20	19
Ipratropium bromide	14	14
Long acting inhaled beta-agonists	7	7
Theophylline	4	1

The patients on treatment with oral prednisone included patients on long term treatment with oral steroids and those who were prescribed prednisone for this exacerbation prior to admission. There was no significant difference between the N-acetylcysteine and placebo groups for any of the concomitant medicines.

All of the subjects completed the study with none being withdrawn early. The rate of change in the Likert scores, lung function and oxygen saturation is shown in Table 4. For the Likert score, FEV_1_, and SaO_2 _the rate of change was greater with placebo than with N-acetylcysteine but none of these differences were statistically significant. Table 5 shows the absolute changes in Likert score, FEV_1 _and SaO_2 _from the beginning to end of the study.

**Table 4:** Slope of least squares regression line

	**N-acetylcysteine **(n = 25)	**Placebo **(n = 25)
**Likert Score**	0.16 (0.42)	0.35 (0.45)
**FEV_1 _% predicted**	0.001 (0.015)	0.019 (0.019)
**Sa0_2_**	0.40 (0.89)	0.88 (1.43)

Mean and standard deviations for slopes of the least square regression lines for the effects of NAC and placebo on Likert scores for breathlessness, FEV_1 _% predicted and SaO_2_. There were no significant differences between NAC and placebo.

**Table 5:** Change in outcome measures from beginning to end of study

	**N-acetylcysteine **(n = 25)	**Placebo **(n = 25)
**Likert Score**	0.7	0.8
**FEV_1 _(litres)**	0.03	0.15
**Sa0_2 _(%)**	1.2	1.8

The average change in Likert score, FEV_1_, VC and SaO_2 _from entry into the study to end of study (discharge or Day 7) are shown.

**Figure 1:**
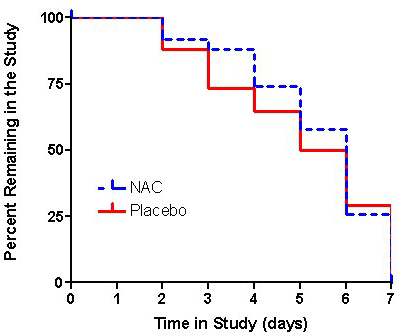
The percentage of patients remaining in the study (i.e. who had not been discharged from hospital) on each day. N-acetylcysteine is shown by a dotted blue line and placebo by a solid red line. The life test procedure of SAS was used to compute nonparametric estimates of the length of stay function by the Kaplan-Meier method. Comparison between these functions was made using the Wilcoxon and log rank tests. Neither the log-rank statistic (p = 0.33) nor the Wilcoxon test (p = 0.30) were significant. The median length of stay was 6.0 days in the NAC group and 5.5 days in the placebo arm.

The Kaplan-Meier analysis showed a similar time course until discharge for the treatment and placebo arms (Figure 1). Neither the log-rank statistic (p = 0.33), which places more weight on longer lengths of stay in hospital, nor the Wilcoxon test (p = 0.30) which places more weight on shorter stays in hospital were significant. The median length of stay was 6.0 in the NAC group and 5.5 in the placebo arm.

Three subjects reported adverse events in each group. One of the subjects treated with N-acetylcysteine reported nausea compared with two of the subjects treated with placebo. There were no serious adverse events.

## Discussion

N-acetylcysteine has been consistently shown to reduce the number of exacerbations of COPD when it is taken for 3 months or more. In contrast we failed to show any benefit when N-acetylcysteine was administered as a treatment for acute exacerbations of COPD. There are a number of possible explanations for the failure to see any benefit.

We cannot exclude the possibility that there was a Type II error and that there is indeed a beneficial effect of N-acetylcysteine in the treatment of acute exacerbations. A larger study would be needed to rule out this possibility but there was less improvement in breathlessness, lung function and oxygen saturation with N-acetylcysteine than with placebo that argues against this explanation.

Another possibility that needs to be considered is that we used too low a dose of N-acetylcysteine and that the concentrations of N-acetylcysteine in the lung were not high enough to exert adequate antioxidant or anti-inflammatory effects. N-acetylcysteine is metabolized to cysteine and this in turn acts as a precursor of reduced glutathione which is an antioxidant [12]. Bridgeman and colleagues studied the effects of administering either 600 mg once daily or 600 mg three times daily [13]. After a single dose of 600 mg, N-acetylcysteine was detected in plasma for 1.5 hours. Plasma cysteine concentrations were also elevated but had returned to baseline by four hours. Glutathione concentrations were variably increased following a single dose of N-acetylcysteine but when N-acetylcysteine was given as 600 mg three times daily (t.i.d) for 5 days the glutathione concentrations were consistently and significantly elevated 12 hours post dose. In this study there was no increase in cysteine or reduced glutathione in either bronchoalveolar lavage fluid or lung tissue (from subjects undergoing pneumonectomy) when the samples were obtained 16–20 hours after the last dose of N-acetylcysteine. In an earlier study, however, reduced glutathione had been shown to be increased in bronchoalveolar lavage fluid 1 to 3 hours after a single dose of 600 mg of N-acetylcysteine [14]. It is likely that the dosing regimen that we used would lead to increases in cysteine and glutathione in both plasma and in the lungs but this may well not have been sustained over the whole of 24 hours. This leaves unanswered the question of whether the changes that did occur in N-acetylcysteine, cysteine and glutathione would have been sufficient to alter the course of the exacerbation. The results of studies where N-acetylcysteine was used as a prophylactic agent to prevent exacerbations of chronic bronchitis and/or COPD would argue that we did use an adequate dose. In these studies doses of N-acetylcysteine between 300 mg b.d. to 600 mg b.d. were effective and the dose that we used in this study is at the upper end of this range. N-acetylcysteine has also been shown to be effective for other indications when it has been used in this dose. Several studies have shown that N-acetylcysteine 600 mg b.d. protects against contrast nephropathy [15,16]. Whether or not a higher dose of N-acetylcysteine would have been any more effective in the treatment of acute exacerbations of COPD can only be answered by conducting additional studies.

In contrast to N-acetylcysteine, prednisone and prednisolone are effective treatments for acute exacerbations of COPD. Davies et al studied 56 patients admitted to hospital with an exacerbation of COPD and found that prednisolone led to a greater improvement in lung function and shortened the hospital stay [17]. Other studies have confirmed the efficacy of corticosteroids in severe exacerbations of COPD [18]. There is evidence of increased numbers of eosinophils in the airways during exacerbations of COPD. Corticosteroids are very effective at suppressing eosinophilic inflammation in the airways and this may account for the benefit seen in exacerbations of COPD. When children with an exacerbation of asthma are treated with prednisone, there is a marked reduction in the concentration of 8-isoprostane in exhaled breath condensate [19]. 8-isoprostane is a marker of oxidative stress. If N-acetylcysteine prevents exacerbations of COPD because it is an anti-inflammatory agent and/or antioxidant, it may be difficult to see additional benefit in established exacerbations of COPD when the patients are also treated with prednisone, which has anti-inflammatory actions and the potential to reduce formation of reactive oxygen species from inflammatory cells.

There have been a number of other studies looking at the effect of mucolytics in acute exacerbations of chronic bronchitis although none of these studies used N-acetylcysteine. Each of these studies has limitations. Langlands treated 27 patients, who had been admitted to hospital with an exacerbation of chronic bronchitis, with bromhexine 8 mg t.i.d. for two weeks (13 patients received bromhexine and 14 received placebo) [20]. In this study lung function was only measured twice a week during the study but the difference between treatments was not statistically significant. Maesen and his colleagues studied 22 patients admitted to hospital with an exacerbation of chronic bronchitis and purulent sputum [21]. All subjects received erythromycin and half were treated with bromhexine. Lung function was not measured but treatment with bromhexine did not influence the bacteriological response to erythromycin. Fimiguerra et al randomized 40 patients who had been admitted to hospital with an exacerbation of chronic bronchitis to treatment with amoxicillin alone (20 patients) or a combination of amoxicillin and domiodol (20 patients) for 10 days [22]. There was a three day washout period before treatment was initiated but it is not clear if this means that patients were in hospital for three days before treatment was started. Lung function was only measured at the beginning and end of treatment but there was no difference between the groups in changes in FEV_1 _and VC. Sputum volumes, however, were greater with the combination of the mucolytic and antibiotic. Ricevuti et al treated 24 patients with an exacerbation of chronic bronchitis [23]. Half of the patients were randomised to a combination of erdosteine and amoxicillin for seven days and the other received amoxicillin alone for the same period of time. Sputum viscosity and temperature resolved significantly more quickly with the combination but lung function was not measured in this study. None of these studies measured lung function on a daily basis and none assessed changes in breathlessness. This makes it difficult to know if treatment with these mucolytics influenced the rate of resolution of the exacerbations. On balance however these studies do not strongly suggest that mucolytics influence the resolution of acute exacerbations of COPD.

## Conclusions

Our study does not suggest that 600 mg b.d. of N-acetylcysteine is effective in the treatment of patients who are admitted to hospital with an acute exacerbation of COPD and who receive concurrent treatment with corticosteroids. In future studies it may be appropriate to use a higher dose of N-acetylcysteine and to compare N-acetylcysteine with placebo in patients with mild exacerbations who do not require treatment with corticosteroids. Consideration could also be given to comparing the effects of N-acetylcysteine with prednisone or prednisolone in patients who would usually be treated with oral corticosteroids.

## Abbreviations

NAC N-acetylcysteine

COPD chronic obstructive pulmonary disease

FEV_1 _forced expiratory volume in one second

VC vital capacity

SaO_2 _oxygen saturation

## Competing interests

The author(s) declare that they have no competing interests.

## Authors' contributions

**PNB **conceived the idea for the study and was responsible for the study design and writing the manuscript. He was also was involved in the conduct of the study and the analysis of the data. **AM-D **was involved in the conduct of the study, in the analysis of the data and with writing the manuscript. **PJP **was involved with the design and conduct of the study. **TEM **and **RPY **were involved with the conduct of the study. All of the authors read and approved the final manuscript.

## Pre-publication history

The pre-publication history for this paper can be accessed here:


